# The first pancreatic neuroendocrine tumor in Li-Fraumeni syndrome: a case report

**DOI:** 10.1186/s12885-020-06723-6

**Published:** 2020-03-30

**Authors:** John G. Aversa, Francine Blumental De Abreu, Sho Yano, Liqiang Xi, Donald W. Hadley, Irini Manoli, Mark Raffeld, Samira M. Sadowski, Naris Nilubol

**Affiliations:** 1 Surgical Oncology Program, Center for Cancer Research, National Cancer Institute, National Institutes of Health, 10 Center Drive, Building 10, Room 4-5952, Bethesda, MD 20892 USA; 2 Laboratory of Pathology, Center for Cancer Research, National Cancer Institute, National Institutes of Health, Bethesda, MD 20892 USA; 3 Medical Genomics and Metabolic Genetics Branch, National Human Genome Research Institute, National Institutes of Health, Bethesda, MD 20892 USA; 4 Human Development Section, Medical Genetics Branch, National Human Genome Research Institute, National Institutes of Health, Bethesda, MD 20892 USA

**Keywords:** Pancreatic neuroendocrine tumor, Li-Fraumeni syndrome, TP53, Hereditary pancreatic neoplasm

## Abstract

**Background:**

Li-Fraumeni syndrome is a cancer predisposition syndrome caused by germline *TP53* tumor suppressor gene mutations, with no previous association with pancreatic neuroendocrine tumors (PNETs). Here we present the first case of PNET associated with Li-Fraumeni syndrome.

**Case presentation:**

This is a 43-year-old female who underwent laparoscopic distal pancreatectomy at age 39 for a well-differentiated grade 2 cystic PNET. When the patient was 41 years old, her seven-year-old daughter was found to have an astrocytoma and a germline *TP53* mutation. While undergoing surveillance with ^68^Gallium-DOTATATE positron emission tomography/computed tomography for her PNET, the patient was found to have a large choroid plexus papilloma in her right temporal lobe. She underwent genetic counseling and testing that identified a germline pathogenic variant in *TP53*, leading to the diagnosis of Li-Fraumeni syndrome. Her PNET had a hemizygous pathogenic *TP53* mutation with loss of the wild-type alternate allele, consistent with loss of heterozygosity and the two-hit hypothesis. She was enrolled in a Li-Fraumeni syndrome protocol and continues surveillance screening with our service.

**Conclusions:**

This is the first PNET reported in association with Li-Fraumeni syndrome. Pancreatic cancer risk is elevated in this syndrome, and our case highlights the need for vigilance in screening for pancreatic neoplasms in these patients.

## Background

Pancreatic neuroendocrine tumors (PNETs) are a rare histologic subtype of pancreatic neoplasm, with an incidence of approximately 0.32/100,000 people in the United States [1, 2]. Although most PNETs occur sporadically, there are four inherited syndromes associated with them: neurofibromatosis type 1 (*NF1* gene; OMIM #162200), von Hippel-Lindau disease (*VHL* gene; OMIM #193300), multiple endocrine neoplasia type 1 (*MEN1* gene; OMIM #131100), and tuberous sclerosis complex (*TSC1* and *TSC2* genes; OMIM #191100 and #191092, respectively). Li-Fraumeni syndrome (LFS) is a rare hereditary cancer predisposition syndrome caused by a heterozygous *TP53* germline mutation [3]. LFS has established associations with breast, brain, adrenal, hematological, and colorectal cancers, along with bone and soft tissue sarcomas, but to date no association with PNET has been reported [4].

Although somatic *TP53* mutations have been identified in various grades of PNETs as well as gastric, small bowel, colorectal, and appendiceal neuroendocrine tumors (NETs), there are no reported cases of NETs associated with a germline *TP53* mutation [2, 5, 6]. *TP53* mutations are commonly found in poorly differentiated neuroendocrine carcinomas [2]. *TP53* is a tumor suppressor gene located on locus 17p13.1 that codes for p53, the most commonly inactivated protein in human cancer [7]. Here we report a patient who initially presented with cystic PNET and later was found to have LFS. We performed next-generation sequencing of the PNET, demonstrating a “second-hit” on *TP53*, which is the first reported manifestation of PNET in association with LFS [8].

## Case presentation

**Fig. 1:**
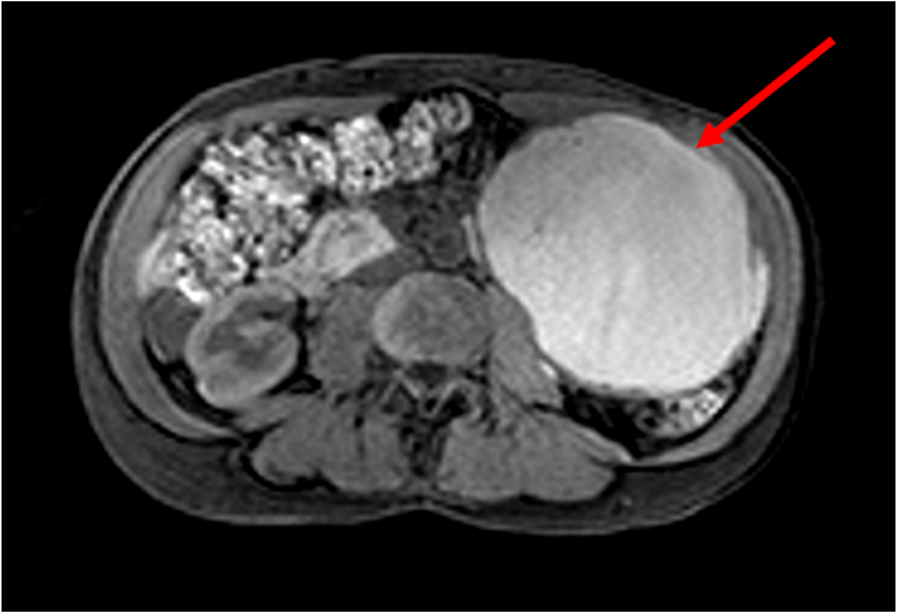
Cross-sectional imaging of cystic PNET. MRI demonstrating a nearly 13 cm cystic pancreatic lesion (identified with the red arrow) that was resected along with a portion of the distal pancreas in 2015. Histopathology showed a World Health Organization grade 2 well-differentiated neuroendocrine tumor

The patient is a 43-year-old female who underwent laparoscopic distal pancreatectomy in 2015 (age 39) after a long-standing history of a large (12.7 × 11 × 10 cm) septated pancreatic cyst with a nodular, thickened wall (Fig. 1). She initially presented with mild discomfort in her left upper abdomen in 2013 (age 37). The physical exam was unremarkable. Imaging studies revealed a large, heterogeneous pancreatic tail cyst, along with a 6 cm heterogeneous left kidney lesion most consistent with angiomyolipoma and a 2.5 cm right liver lesion consistent with hemangioma. Surgical resection of the pancreatic cyst was recommended, but the patient instead elected to undergo transgastric endoscopic fine-needle aspiration at an external facility in 2014. The cyst recurred (found on follow-up imaging), and she then underwent laparoscopic distal pancreatectomy in 2015.

Pathologic examination was consistent with a World Health Organization (WHO) well-differentiated grade 2 PNET (the Ki-67 proliferation index was 3.5%, and the mitotic rate was < 1/50 high-powered fields). The tumor cells stained positive for chromogranin and synaptophysin. The patient continued surveillance visits and imaging, with no evidence of recurrence.

In 2017, her seven-year-old daughter was diagnosed with a WHO grade 3 isocitrate dehydrogenase wild-type intracranial astrocytoma with a *TP53* somatic mutation identified in the tumor. A germline pathogenic mutation in *TP53* was also identified, but exact variant information was not available from the testing laboratory. Parental testing was not performed at the time. Shortly after diagnosis, she died from tumor progression.

**Fig. 2:**
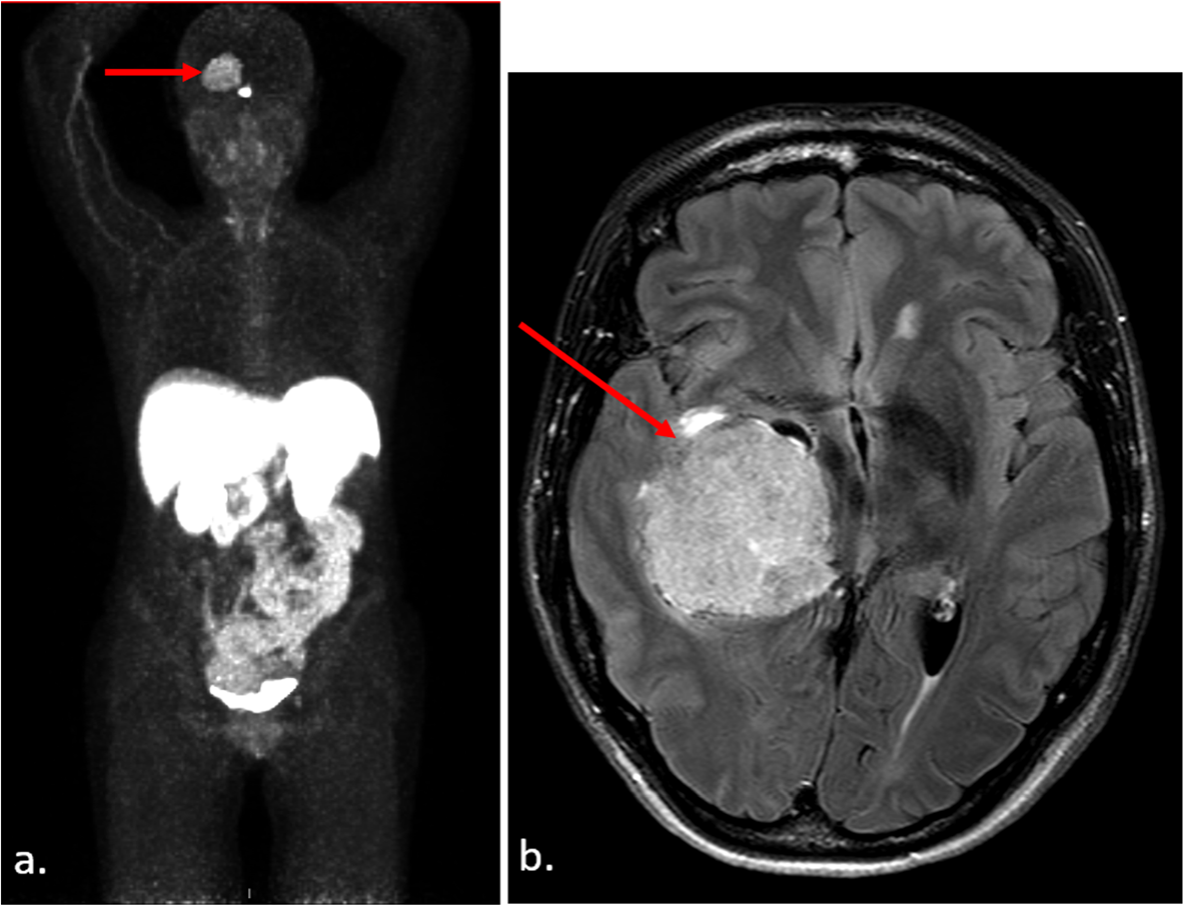
^68^Gallium-DOTATATE PET/CT study revealing a right-sided, avid intracranial mass. **a.** Planar whole-body maximum-intensity projection, with red arrow identifying the intracranial mass. **b.** Fluid-attenuated inversion recovery (FLAIR) post-contrast brain MRI demonstrating a 5.3 × 3.3 × 4.3 cm enhancing intraventricular mass (identified with red arrow) in the temporal horn of the right ventricle, which was associated with near-complete compression of the right lateral ventricle and 6 mm midline shift at the level of the foramen of Monro

In 2018 (age 42), the patient underwent a ^68^Gallium-DOTATATE positron emission tomography/computed tomography (PET/CT) scan for PNET surveillance, which revealed a large right temporal lobe mass that was further characterized on brain magnetic resonance imaging (MRI) to represent a large (5.3 × 3.3 × 4.3 cm) enhancing intraventricular mass causing mass effect and midline shift (Fig. 2).

In light of this finding and her daughter’s history, the patient was evaluated by neurosurgical and genetic counseling teams. She underwent craniotomy and resection for the intracranial lesion. Pathologic examination showed that the mass was a WHO grade 1 choroid plexus papilloma, that stained positive for synaptophysin, glial fibrillary acidic protein, cell adhesion molecule 5, p53, and cytokeratin 7. Table 1 shows a timeline of major events in the patient’s care.

**Table 1:** Important dates in the case

Date	Event
8/2013	Began to develop symptoms of abdominal discomfort
3/2014	MRI showing large cystic mass in pancreatic tail
8/2014	Endoscopic aspiration of pancreatic tail cyst
3/2015	Laparoscopic distal pancreatectomy/splenectomy
8/2017	Patient’s daughter diagnosed with astrocytoma
10/2018	Underwent craniotomy and resection for right temporal lobe choroid plexus papilloma detected on PET/CT
2/2019	Genetic testing detected a germline *TP53* mutation

The patient’s paternal ancestry is Russian, and her maternal ancestors are non-Ashkenazi Russian Jewish. Her maternal grandmother’s fraternal twin sister died at age 65 of a rare gastrointestinal (GI) cancer, and her paternal grandmother had uterine cancer in her 50s. The detailed history of the patient’s other family members, particularly on her father’s side, is unknown.

The patient underwent genetic testing in a Clinical Laboratory Improvement Amendments–certified molecular genetics laboratory (Invitae, San Francisco, CA) and was found to carry a pathogenic germline *TP53* mutation, namely a cytosine-to-thymine transition at codon 1009, leading to an arginine-to-cysteine substitution (c.1009C > T, p.R337C). Initial surveillance exams and studies were performed according to MD Anderson Cancer Center’s LFS Education and Early Detection Adult Screening Guidelines [9].

Our patient’s care plan includes a yearly whole-body MRI, a yearly breast thermogram (the patient refused a mammogram) and breast MRI, a yearly brain MRI, abdominal and pelvic ultrasounds every 6 months, esophagogastroduodenoscopy and colonoscopy every two to 5 years, and yearly labs. Additionally, the patient will undergo a clinical breast exam every 6 months and a yearly skin exam. She will continue with a yearly abdominal MRI for PNET and angiomyolipoma surveillance.

Surveillance detected no conclusive evidence of additional neoplasms, but it revealed several indeterminate osseous lesions that are currently being monitored.

Paraffin-embedded PNET tissue from 2015 was assayed for a profile of common somatic mutations (Oncomine Comprehensive Assay v3, Thermo Fisher Scientific, Carlsbad, CA) and found to have a hemizygous pathogenic *TP53* mutation at codon 1009 (NM_000546.5: c.1009C > T, p.R337C). Variant allele frequency was 93%, consistent with loss of the wild-type alternate allele (loss of heterozygosity) as a “second hit” in the tumor suppressor gene.

## Discussion and conclusions

Here we report the first PNET in association with LFS. Next-generation sequencing reveals a hemizygous pathogenic *TP53* mutation (p.R337C) consistent with a loss of heterozygosity at the locus. This represents a novel finding that expands the clinical spectrum of LFS. Although *TP53* somatic mutations are frequently found in neuroendocrine carcinoma, they do not commonly appear in well-differentiated NET with prolonged disease-free survival, such as in this patient [2, 5, 6].

Prior studies demonstrate somatic *TP53* mutations in all grades of NET from various primary sites, with high-grade tumors more commonly harboring the mutation in both intra-abdominal and extra-abdominal NETs. Overall, more than half of poorly differentiated GI NECs have somatic *TP53* mutations, while 0–11% of moderately and well-differentiated GI NETs harbor the mutation [2, 5, 10, 11]. Similarly, for pulmonary NETs, more than 60% of small cell lung cancer and large cell neuroendocrine carcinoma have *TP53* mutations, and 0–11% of well-differentiated grade 1 and 2 pulmonary NETs have been shown to have *TP53* mutations [12, 13].

None of these prior studies that reported somatic mutations in NETs included germline mutation sequencing for their study cohorts. Our report demonstrates not only the next-generation sequencing of our PNET verifying a hemizygous *TP53* mutation and loss of the wild-type allele, but also shows our patient’s *TP53* germline mutation with other clinical manifestations consistent with LFS.

Although the prevalence and the risk of developing PNET in patients with LFS is unknown, this patient population has a known predisposition for pancreatic adenocarcinoma at a rate of approximately seven times that of the general population [14]. Our patient had no additional pathogenic mutations detected by the Oncomine Comprehensive Assay, and we postulate that a “second-hit” somatic *TP53* mutation in the setting of an existing germline *TP53* mutation drove the development of her PNET. However, it is possible that there are additional pathogenic somatic mutations that were not included in this assay.

In the setting of a new PNET diagnosis, current guidelines advocate for either recommendation (in the case of *VHL*, *TSC*, and *NF1*) or consideration (in the case of *MEN1*) of genetic testing when a patient has a personal and/or family history compatible with the known associated syndromes [15]. Similar advice is reasonable here, as the majority of PNETs are sporadic and this is the first described case associated with LFS.

Our patient’s angiomyolipoma in the context of her LFS diagnosis warrants further consideration. She follows up with the urologic surgery service for surveillance, and although she and the service have discussed a biopsy to determine the presence of malignant features, she has opted for imaging surveillance at this time. Although angiomyolipomas are normally benign lesions, there have been reports of malignant angiomyolipoma in LFS patients, which emphasizes the importance of surveillance and, potentially, the need for intervention [16].

The current clinical criteria (Chompret criteria) to identify suspected LFS patients who are candidates for genetic testing centers around presentation according to family history, multiple primary tumors, rare tumors, or early-onset breast cancer [17]. Familial criteria for testing include typical LFS-associated tumors (osteosarcoma, soft tissue sarcoma, central nervous system tumors, premenopausal breast cancer, and adrenocortical carcinoma) diagnosed in the proband at an age younger than 46 years, as well as at least one first- or second-degree relative who presents with an LFS-related tumor before age 56 or who has multiple tumors [17]. The multiple primary tumor criteria require that the proband presents with multiple primary tumors (except breast cancer), with two of the tumors belonging to the LFS tumor spectrum, the first of which must appear before age 46 [17]. The rare tumor criteria include any patient with adrenocortical carcinoma, choroid plexus tumor, or the embryonal anaplastic subtype of rhabdomyosarcoma [17]. Patients who present with breast cancer before 31 years of age are recommended to undergo *TP53* germline mutation testing as well [17]. Although most PNETs present in patients 50 years old or above, it is not uncommon for patients to present at a younger age [18, 19]. Only in conjunction with additional findings or a family history suggestive of LFS would we recommend testing for a *TP53* germline mutation in new diagnosis of PNET.

Our findings in this case would not change the surveillance program for pancreatic neoplasm screening in LFS (yearly whole-body MRI under many LFS screening protocols). We recommend a dedicated abdominal MRI in LFS patients to increase sensitivity in the detection of pancreatic neoplasms. This is also commonly recommended in LFS screening guidelines, including the MD Anderson high-risk pancreatic cancer screening guidelines [20]. In this particular situation, where a patient has manifested PNET in association with LFS, we recommend a dedicated abdominal MRI every 6 months for 2 years after resection, and yearly thereafter if there remains no evidence of disease. Interpreting the relevance of our patient’s development of PNET to her specific *TP53* mutation (p.R337C) is difficult, as genotype–phenotype relationships in LFS are not fully characterized [17, 21, 22]. Prior analyses have categorized *TP53* pathogenic variants by genetic location and by predicted structural and/or functional properties of mutant proteins. Missense mutations affecting residues belonging to loops 2 and 3 of the p53 protein were found to be associated with the development of brain tumors [14, 23]. Missense mutations affecting residues outside of the loop-sheet-helix motif that binds the major groove of target DNA in p53 were found to be associated with the development of adrenocortical carcinomas [14, 23].

Attempts have been made to classify *TP53* mutation pathogenic variant with LFS phenotype. Ideally, this would provide insight for more targeted screening for specific malignancies in these carriers. Although genotypic variation has not been shown to correlate with the development of particular tumors, dominant-negative missense *TP53* mutations leave their carriers susceptible to cancer at earlier ages and at higher levels of clinical severity than those with non-missense mutations [17]. Despite the limitations of our finding in the broad scope of LFS, it is important to consider when developing more targeted screening protocols for specific LFS genotypes.

Germline *TP53* mutations most commonly occur within the DNA-binding domain, leading to the production of a dysfunctional p53 protein [24]. Our patient’s particular *TP53* mutation is relatively uncommon and has initially been implicated in LFS and Li-Fraumeni-like syndromes [25]. A cytosine-to-thymine transition at nucleic acid 1009 leads to a missense arginine-to-cysteine transition at amino acid 337 (p.R337C) within exon 10. This results in p53 alteration along its basic C-terminus, leading to a reduction in overall functioning p53 levels; tetramerization fraction; and, therefore, its DNA-binding ability and transactivation [26, 27]. The p.R337C germline mutation has been seen in cohorts that tend to develop brain tumors, osteosarcoma, rhabdomyosarcoma, breast cancer, and childhood adrenocortical carcinoma [25, 27–31].

A prior analysis established three age-based categories along a “cancer spectrum” of LFS [32]. Classification included tumors belonging to the “childhood phase” (adrenocortical carcinoma, choroid plexus carcinoma, rhabdomyosarcoma, and medulloblastoma), “early adulthood phase” (breast cancer, osteosarcoma, leukemia, astrocytoma, glioblastoma, colorectal cancer, and other sarcomas [malignant fibrous histiocytoma, liposarcoma, leiomyosarcoma]), and “late adulthood phase” (pancreatic and prostate cancer) [32]. Genotypic variation in LFS has been best correlated with a phenotype in Brazilian families that harbor the more common *TP53* germline mutation that features a transition from guanine to adenine and a subsequent arginine-to-histidine transition (c.1010G > A, p.R337H), rendering carriers particularly susceptible to the development of adrenocortical carcinoma [31].

This patient’s case represents the first reported PNET in association with a germline *TP53* mutation, highlighting the importance of screening for pancreatic neoplasms in the setting of LFS. The p.R337C mutation is an uncommon pathogenic variant of *TP53* in LFS, and much remains to be described about its associated cancers. To aid patient surveillance programs, further studies are warranted to elucidate a genotype–phenotype correlation and the other molecular mechanisms associated with LFS phenotypes. This case report was prepared following the CARE guidelines and methodology [33].

## Data Availability

Supporting data can be found in the Clinical Research Information System, which is the electronic medical record at the National Institutes of Health. These data are not available publicly due to patient privacy restrictions but are available from the corresponding author upon reasonable request.

## Abbreviations

c.1009C > T: Mutation at codon 1009 cytosine to thymine
c.1010G > A: Mutation at codon 1010 guanine to adenine
CT: Computed tomography
FLAIR: Fluid-attenuated inversion recovery
GI: Gastrointestinal
LFS: Li-Fraumeni syndrome
MEN1: Multiple endocrine neoplasia type 1
MRI: Magnetic resonance imaging
NET: Neuroendocrine tumor
NF1: Neurofibromatosis type 1
p.R337C: Mutation at amino acid 337 arginine to cysteine
p.R337H: Mutation at amino acid 337 arginine to histidine
PET: Positron emission tomography
PNET: Pancreatic neuroendocrine tumor
TSC: Tuberous sclerosis complex
VHL: Von Hippel-Lindau
WHO: World Health Organization
